# Gambling and COVID-19: Initial Findings from a UK Sample

**DOI:** 10.1007/s11469-021-00545-8

**Published:** 2021-06-04

**Authors:** Steve Sharman, Amanda Roberts, Henrietta Bowden-Jones, John Strang

**Affiliations:** 1 Addictions Department, Institute of Psychiatry, Psychology and Neuroscience, King’s College London, 4 Windsor Walk, Camberwell, London, SE5 8AF UK; 2 School of Psychology, University of East London, Water Lane E15 4LZ, Stratford, UK; 3 School of Psychology, University of Lincoln, Brayford Pool, Lincoln, LN6 7TS UK; 4 Division of Psychology and Language Sciences, UCL, 26 Bedford Way, Bloomsbury, WC1H 0AP, London, UK; 5 Department of Psychiatry, University of Cambridge, Herchel Smith Building for Brain & Mind Sciences, Forvie Site, Robinson Way, Cambridge, CB2 0SZ UK; 6 National Problem Gambling Clinic, 69 Warwick Road, London, SW5 9BH UK

**Keywords:** Gambling, Disordered gambling, COVID-19, Lockdown, Behavioural addiction

## Abstract

In response to the COVID-19 pandemic, the UK Government placed society on ‘lockdown’, altering the gambling landscape. This study sought to capture the immediate lockdown-enforced changes in gambling behaviour. UK adults (*n* = 1028) were recruited online. Gambling behaviour (frequency and weekly expenditure, perceived increase/decrease) was measured using a survey-specific questionnaire. Analyses compared gambling behaviour as a function of pre-lockdown gambling status, measured by the Brief Problem Gambling Scale. In the whole sample, gambling participation decreased between pre- and during-lockdown. Both gambling frequency and weekly expenditure decreased during the first month of lockdown overall, but, the most engaged gamblers did not show a change in gambling behaviour, despite the decrease in opportunity and availability. Individuals whose financial circumstances were negatively affected by lockdown were more likely to perceive an increase in gambling than those whose financial circumstances were not negatively affected. Findings reflect short-term behaviour change; it will be crucial to examine, at future release of lockdown, if behaviour returns to pre-lockdown patterns, or whether new behavioural patterns persist.

Gambling is a common activity in the UK, and throughout the world. The Health Survey for England (HSE, 2018) reported that 54% of adults in the UK had gambled in the preceding 12 months. Using the Problem Gambling Severity Index (PGSI, Ferris & Wynne, 2001), the survey estimated that 3.6% of the population experience some degree of gambling-related harm (e.g. PGSI score of ≥1), and that 0.4% of the population experience problem gambling (e.g. a score of ≥8 on the PGSI) (HSE, 2018). A more recent study from 2020 estimated that approximately 61% of the UK adult population (16+) had gambled in the past 12 months and reported that the prevalence of problem gambling in the UK was 3% (GambleAware, 2020). When considering sub-clinical levels of gambling related harm, a further 10% of the population are at risk of gambling-related harm (GambleAware, 2020). Prevalence rates are significantly higher in the GambleAware data than the HSE data; analysis of differences is highlighted within the GambleAware report, which concludes that the difference is perhaps due to different data collection methodologies, and that the true prevalence is likely to lie somewhere between the two estimates.

The UK currently has a permissive gambling environment. Land-based gambling is widely available in a variety of premises including bookmakers, casinos, bingo halls, motorway services, supermarkets and corner shops. The proliferation of online gambling and the global nature of sporting competitions ensure that betting is available 24 h a day, and that anyone with a smartphone and an internet connection has unfettered access to gambling. The wide availability and accessibility make it possible to gamble on almost anything, at any time (Gainsbury et al., 2015).

The UK gambling landscape was significantly altered on the 23rd March 2020, when the UK Prime Minister announced a range of measures designed to stem the spread of the COVID-19 pandemic and included the closure of all non-essential retail outlets, including land-based gambling establishments. Referred to by the UK media as ‘lockdown’, the order was initially put in place for 3 weeks but only gradually relaxed 3 months later at the end of June 2020 (Coronavirus: lockdown to be relaxed in England as 2m rule eased, n.d.). Whilst the ‘lockdown’ measures were put in place to arrest the spread of COVID-19, the resulting social, economic and situational environments generated have been conducive to enforcing changes in addictive behaviours (Marsden et al., 2020).

The impact of lockdown has been shown to be potentially harmful for gamblers (Håkansson et al., 2020; van Schalkwyk etal., 2020). A range of risk factors have been previously identified for disordered gambling (for reviews, see Dowling et al., 2017; Sharman et al., 2019), and some of these factors may be exacerbated in the lockdown environment including boredom (Blaszczynski et al., 1990; McCormack et al., 2014; Mercer & Eastwood, 2010) and social isolation (Bergh & Kühlhorn, 1994; Gill & McQuade, 2012; King et al., 2010; McMillen et al., 2007; Trevorrow & Moore, 1998).

Furthermore, the measures enforced by the UK Government in response to the COVID-19 outbreak have placed a significant financial strain on a large number of households, e.g. through furlough or job loss; (Osborne, 2020). Evidence suggests that financial insecurity can negatively influence psychological well-being (as measured by self-esteem, depression and anxiety), increase the likelihood of risky financial decision (Weinstein & Stone, 2018) and can also have an adverse influence on economic decision making (Haushofer & Fehr, 2014). When viewed in a gambling context, the effect of financial insecurity on approach to risk can lead to more risky gambling (Dussault et al., 2011). Likewise, lower household income has been shown to be associated with problem gambling, even after controlling for socio-demographic variables (Orford, 2004).

The impact of COVID-19 and the subsequent lockdown dramatically altered both the availability and accessibility of gambling. Many major sporting events across Europe and the world were either cancelled or postponed, including major UK sports and events such as the Premier League, and the Wimbledon tennis championships. Additionally, in the UK, land-based gambling opportunities were severely restricted as casinos and bookmakers were closed. Conversely, supermarkets remained open, allowing the sale of lottery tickets and scratchcards to continue.

Research on the influence of lockdown on gambling behaviour is scarce but some reports offer some preliminary findings. Using online operator data across four European countries, one study showed that the migration of sports bettors to online casino games was absent, and that there was a decrease in money wagered by sports bettors. Findings indicated that when there was no sport to bet on, sports gamblers spent less money on gambling, rather than simply changing to gambling on other available methods such as casino games (Auer et al., 2020). In Sweden, an online study found that only a minority of participants reported increases in gambling, but this group also reported higher gambling problems (Håkansson, 2020). In the UK, the data collated by the Gambling Commission found that overall, the number of ‘active player accounts’ decreased between March and April 2020, indicating a general trend of less people gambling (online), but with more ‘engaged’ gamblers (those who have gambled on three or more activities in the past 4 weeks) spending more time and money gambling (Gambling Commission, 2020). The Gambling Commission does not explicitly identify more engaged gamblers as problem gamblers, yet, both higher frequency of gambling (O'Mahony & Ohtsuka, 2015), and higher gambling spend (Brosowski et al., 2015; Currie et al., 2009) are associated with disordered gambling. Disordered gamblers are known to contribute a disproportionately large amount of money and time spent gambling, a phenomenon observed both in the UK (Orford et al., 2013), and worldwide (Fiedler, 2011; Fiedler et al., 2019; Miller & Singer, 2015; Tom et al., 2014).

The nature of the social climate in the UK created by the implementation of lockdown means the short- and longer-term influences on gambling behaviour are as yet, not well understood. This study aims to provide an initial analysis of gambling behaviour change during the first phase of lockdown, in the UK. Specially, the study aims to:

1. Examine frequency of gambling, and money spent gambling pre- and during-lockdown
2. Investigate frequency of gambling and money spent gambling pre- and during-lockdown as a function of pre-lockdown gambling risk level
3. Investigate the relationship between gambling behaviour and the negative financial effects of lockdown.

## Methods

### Recruitment and Participants

Participants were recruited through Prolific Academic, an online recruitment tool (www.prolific.co). Participants recruited from Prolific Academic have been found to be more naïve and less dishonest than those recruited from MTurk, and to produce higher quality data than the alternative platform CrowdFlower (Peer et al., 2017). In the current climate, it is increasingly important to be able to collect data remotely; crowdsourcing is a common data collection tool in the wider psychology field (Buhrmester et al., 2011; Huff & Tingley, 2015), and has more recently been utilised in gambling studies (Mishra & Carleton, 2017; Schluter et al., 2018), although results should be interpreted with caution (Pickering & Blaszczynski, 2021).

To maximise responses, the only eligibility criteria specified were that participants were required to be a current UK resident, and were engaged in some form of social exclusion. Thirteen participants were excluded as they were not engaged in any form of social exclusion, resulting in a final sample of 1028 participants (72.1% female; age *M* = 33.19, SD = 11.66, range 18–73). Age did not differ significantly between males (*M* = 32.68, SD 12.26) and females (M = 33.46, SD 11.45) (*t* (990) = 0.94, *p* = 0.35).

All participants were engaged in some level of measures to prevent the spread of COVID-19, either social distancing, social isolation or social shielding. For convenience, the term social distancing is used henceforth to encapsulate all levels, except where a more specific term is deemed appropriate. In the whole sample (*n* = 1028), 49.1% (*n* = 505) had gambled in the 12 months preceding lockdown; 523 were Non-Gamblers (NG; *n* = 50.9%), 362 were Non-Problem Gamblers (NPG; 35.2%) and 143 were Potential Problem Gamblers (PPG; 13.9%). Participants were most commonly social distancing in a household with 2–3 other people (40.5%), and least commonly distancing alone (15%). Most were distancing with family (76.46%); 76.17% had been distancing for between 2 and 4 weeks, at the time of survey completion. Prior to COVID-19, participants had most commonly been employed (62.84%), and 21.4% indicated that their employment status had changed since the outbreak of COVID, with the most common change amongst those who status had changed reported as being furloughed (47.7%).

### Measures

Problem gambling status was measured using the Brief Problem Gambling Screen (BPGS-5, Volberg & Williams, 2011). The BPGS consists of five yes/no binary questions, and was used due to its brevity, and robust psychometric properties. Model development indicated that a five-item model demonstrated high specificity (99.9%) and sensitivity (90.8%), and greater clarification accuracy than other two-, three- or four-item models (ibid*)*. A score of 1 or more indicates potential problem gambling, and a need for further assessment (Stinchfield et al., 2012). The BPGS has been used in previous gambling research (McCarthy et al., 2021), and compares favourably to other brief gambling screening tools, reported as being ‘the only instrument displaying satisfactory classification accuracy in detecting any level of gambling problem’ (Dowling et al., 2018). The BPGS was used to group participants into non-gambler (no gambling in the preceding 12 months), non-problem gambler (gambled in preceding 12 months but scored 0 on BPGS) and potential problem gambler (gambled in previous 12 months and scored ≥1 on BPGS) groups for subsequent analysis.

The study utilised a retrospective self-report longitudinal design. The study was programmed in Qualtrics, an online data collection tool and administered through prolific academic. Data were collected in a single session and questioned behaviours covering two time periods; the first time period refers to a specified period prior to the Government-recommended social distancing measures and is henceforth referred to as pre-lockdown. Questions also asked participants to self-report behaviour since being asked to socially isolate, referred to henceforth as during-lockdown (i.e. since the Government announcement on 23rd March).

Participants were asked questions relating to social distancing: what type of distancing, how many people they were distancing with and how long they had been distancing for. Participants were also asked their pre-lockdown employment status, and whether that status had changed in lockdown. Gambling behaviour was measured with survey-specific questions asking participants to detail their gambling behaviour. Questions referenced gambling pre-lockdown, and during-lockdown, and asked about frequency of gambling, and gambling expenditure both as a raw amount, and a proportion of income, and the reasons for gambling. Participants were also asked whether they perceived their gambling had increased, decreased or stayed the same in lockdown.

### Procedure

Data were collected in April 2020. Participants were invited to partake in the study through having a registered Prolific Academic account. Participants gave online consent, and were paid £6.28 p/h, pro-rata for estimated study completion time, resulting in a payment of £1.78 per participant, considered ‘fair’ by Prolific Academic. The study protocol was approved by the School of Psychology Research Committee at the University of Lincoln ref.: 2020–2392, and the University of East London Research Ethics Committee, ref.: ETH1920–0207.

### Data Analysis

Gambling behaviour data regarding frequency and expenditure was compared pre- and during-lockdown across the whole sample, within current gamblers and as a function of BPGS score. The McNemar test for 2 × 2 binomial contingency tables was used when comparing between time periods (i.e. comparing pre- and during-lockdown). Uncorrected *p* values for the McNemar tests calculated via a custom macro (http://www.how2stats.net/search?q=mcnemar) are reported as the Yates correction is considered too conservative (Greenwood, 1996). Between-group data from gambling frequency and spend variables was analysed using chi-squared models or Fisher’s exact test when compared within a specific timeframe (i.e. between groups pre- or during-lockdown). Adjusted z score residuals were used to identify post hoc differences in chi-squared models using the appropriately adjusted *p* value (Beasley & Schumacker, 1995). Cramer’s V was reported as a measure of effect size (0.1, small effect size, 0.3 medium, 0.5 large). Bonferroni corrections were applied to alpha rates as appropriate. All analysis was run in SPSS 26.

## Results

### Frequency: Whole Sample

When comparing the whole sample, participants were less likely to report gambling 1–2 times per month (McNemar χ^2^ (1) = 39.27, *p* < 0.001), and less frequently than 1–2 times per month (McNemar χ^2^ (1) = 147.92, *p* < 0.001) during-lockdown, than pre-lockdown. The differences in number of participants who gambled daily (McNemar χ^2^ (1) = 1, *p* = 0.32), 2–6 times per week (McNemar χ^2^ (1) = 0.91, *p* = 0.34), and once per week (McNemar χ^2^ (1) = 1.16, *p* = 0.28) did not differ between pre- and during-lockdown.

### Frequency: Current Gamblers

Within current gamblers (who had gambled in the 12 months preceding lockdown), there was a decrease in the number of gamblers who gambled 1–2 times per month (McNemar χ^2^ (1) = 43.61, *p* < 0.001) between pre- and during-lockdown. The number of current gamblers who gambled daily (McNemar χ^2^ (1) = 0.67, *p* = 0.41), 2–6 times per week (McNemar χ^2^ (1) = 3.57, *p* = 0.06), and once per week (McNemar χ^2^ (1) = 2.78, *p* = 0.09) did not differ between pre- and during-lockdown. Additionally, a further 276 participants who had gambled in the 12 months preceding lockdown had not gambled at all in lockdown.

### Frequency: By Gambling Risk Status

Participants in the PPG group gambled at different frequencies pre-lockdown than the NPG group (χ^2^ (4) = 15.81, *p* = 0.003, Cramer’s *V* = 0.28). Post hoc analyses indicate that the PPG group were more likely to report gambling 2–6 times per week (*p* = 0.027) and were less likely to report gambling less than 1–2 times per month (*p* = <0.001). Analysis of between group during-lockdown gambling shows that the NPG and the PPG group did not differ overall on gambling frequency (χ^2^ (4) = 7.76, *p* = 0.10, Cramer’s *V* = 0.28).

Gambling frequency changes were also measured within gambler groups between pre- and during-lockdown. The NPG group were less likely to report gambling 1–2 times per month (McNemar χ^2^ (1) = 24, *p* < 0.001) and less frequently than 1–2 times per month (McNemar χ^2^ (1) = 137.2, *p* < 0.001) during-lockdown, than pre-lockdown. The NPG group did not differ between pre- and during-lockdown on frequencies of gambling daily (McNemar χ^2^ (1) = 0.91, *p* = 0.76), 2–6 times per week (McNemar χ^2^ (1) = 3.27, *p* = 0.071) or once per week (McNemar χ^2^ (1) = 0, *p* = 1). Furthermore, within the NPG, 208 participants (57.5%) had moved from sometimes gambler (i.e. reported some frequency of gambling) to non-gambler.

**Fig. 1:**
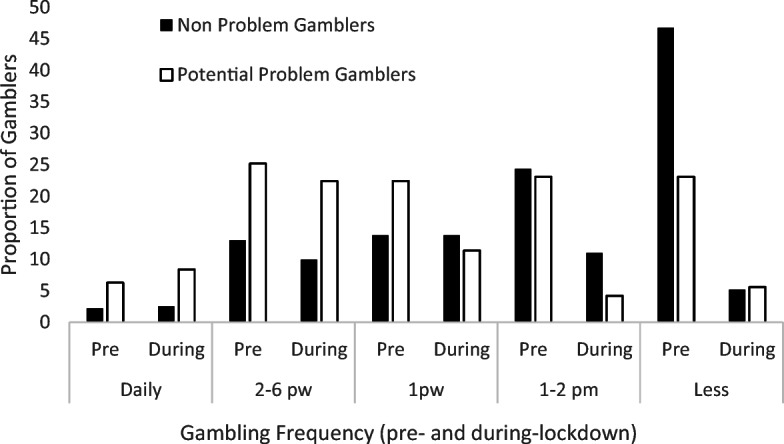
Gambling frequency pre- and during-lockdown by Gambler risk group

The PPG group were less likely to report gambling once per week (McNemar χ^2^ (1) = 9.78, *p* = 0.002), 1–2 time per month (McNemar χ^2^ (1) = 22.09, *p* < 0.001), and less frequently than 1–2 times per month (McNemar χ^2^ (1) = 17.46, *p* < 0.001) during lockdown, than pre-lockdown. The PPG group did not differ between pre-and during-lockdown on frequencies of gambling daily (McNemar χ^2^ (1) = 0.69, *p* = 0.41), or 2–6 times per week (McNemar χ^2^ (1) = 0.62, *p* = 0.43) (Fig. 1).

### Expenditure: Whole Sample

When comparing the whole sample, participants were less likely to report a weekly gambling spend of £1–£25 (McNemar χ^2^ (1) = 174.22, *p* < 0.001), and of £26–£50 (McNemar χ^2^ (1) = 6.12, *p* = 0.012). The difference in the number of participants who reported a weekly spend of £51–£100 (McNemar χ^2^ (1) = 1.58, *p* = 0.21), £101–£200 (McNemar χ^2^ (1) = 0.82, *p* = 0.37) and over £200 (McNemar χ^2^ (1) = 1.6, *p* = 0.21), did not differ between pre- and during-lockdown.

### Expenditure: Current Gamblers

Within current gamblers (who had gambled in the 12 months preceding lockdown), participants were less likely to report spending between £1 and £25 (McNemar χ^2^ (1) = 204.25, *p* < 0.001), and between £26 and £50 (McNemar χ^2^ (1) = 9.62, *p* = 0.002) per week during lockdown, than pre-lockdown. The difference in the number of current gambling participants who reported a weekly spend of £51–£100 (McNemar χ^2^ (1) = 2.79, *p* = 0.09), £101–£200 (McNemar χ^2^ (1) = 0.82, *p* < 0.37) and over £200 (McNemar χ^2^ (1) = 1.6, *p* = 0.21) did not differ between pre- and during-lockdown.

### Expenditure: By Gambling Risk Status

Participants in the PPG group reported a different pattern of expenditure pre-lockdown than the NPG group (Fisher’s Exact Test = 49.3, *p* = <0.001, Cramer’s *V* = 0.46). Post hoc analyses indicate that the PPG group were less likely to spend £1–£25 per week (*p* < 0.001), were more likely to spend £26–£50 per month (*p* = 0.01) and £101–£200 per month (*p* < 0.001). Patterns of expenditure also varied between the NPG and PPG groups during-lockdown (Fishers Exact Test = 13.58, *p* = 0.004, Cramer’s *V* = 0.38). Post hoc analyses indicate that the NPG group were more likely to report spending between £1 and £25 per week (*p* = 0.009).

Weekly gambling expenditure was also measured within gambler groups between pre- and during-lockdown. The NPG group were less likely to report spending between £1 and £25 per week on gambling during-lockdown than pre-lockdown (McNemar χ^2^ (1) = 180.94, *p* < 0.001). The NPG group did not differ between pre- and during-lockdown on the number of participants spending £26–£50 (McNemar χ^2^ (1) = 1.47, *p* = 0.23), £51–£100 (McNemar χ^2^ (1) = 1, *p* = 0.32) or over £200 (McNemar χ^2^ (1) = .20, *p* = 0.65).

**Fig. 2:**
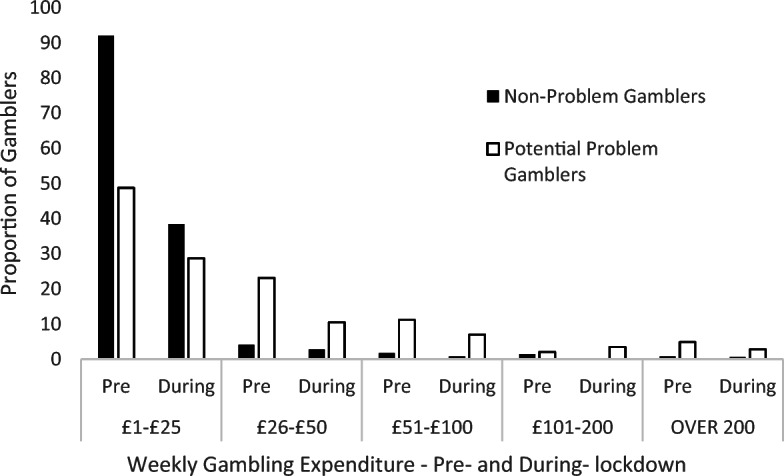
Weekly gambling expenditure pre- and during-lockdown by Gambler risk group

The PPG group were less likely to report spending between £1 and £25 (McNemar χ^2^ (1) = 27.6, *p* < 0.001), and between £26 and £50 (McNemar χ^2^ (1) = 8.53, *p* = 0.004) during-lockdown, compared to pre-lockdown. The PPG group did not differ between pre- and during-lockdown on the number of participants spending £51–£100 (McNemar χ^2^ (1) = 1.8, *p* = 0.18), £101–£200 (McNemar χ^2^ (1) = .66, *p* = 0.41) or over £200 (McNemar χ^2^ (1) = 1.8, *p* = 0.18) (Fig. 2).

### Employment Status and Gambling

Overall, 18% (*n* = 185) of participants reported a change in employment status that could result in a decrease in income (Lost job (*n =* 34, 3.3%), reduced hours (*n =* 17, 1.7%), furloughed (*n =* 105, 10.2%) or loss of work (freelance/self-employed) (*n =* 29, 2.8%). Change in gambling behaviour in those that had gambled during-lockdown was analysed as a function of whether the participant’s employment status had changed during lockdown. The chi-squared model was significant (χ^2^ (2) = 10.14, *p* = 0.006), indicating a significant change in category distribution. Post hoc analysis of adjusted z score residuals indicate that those whose employment status had changed, resulting in a likely decrease in income, were more likely to feel their gambling had increased (*p* = 0.006).

## Discussion

The current study provides initial data on the influence of the Government-enforced social isolation in response to the COVID-19 pandemic on gambling behaviour in the UK. Results are discussed below.

These results indicate that for the majority of the sample, frequency of gambling decreased in the first month of lockdown. It is interesting to note that the only group that did not report a reduction in gambling frequency were those in the PPG group who reported gambling daily, or between 2 and 6 times per week, pre-lockdown. Accessibility of gambling is an important factor in gambling engagement (Meyer et al., 2019); the ubiquitous nature of online gambling ensured that gambling remained available in lockdown. Despite the accessibility of online gambling and the consistent availability of lottery products in shops, other methods of gambling access such as bookmakers’ shops and casinos were closed for the study period, and almost all sporting events were postponed or cancelled, thus reducing gambling opportunity. Such factors may have contributed to the overall drop in gambling, and the reduction in gambling frequency of less engaged gamblers. It is only those who were most engaged with gambling pre-lockdown that maintained their gambling engagement into lockdown despite limited availability of gambling.

Gambling spend also appeared to have either decreased or stayed the same in the first month of lockdown. It is feasible that individuals spending at the lower end of the scale are more casual gamblers, and that restrictions on gambling availability reduced gambling spend, whilst those with a higher engagement with gambling, as evidenced by higher pre-lockdown spend, maintained expenditure levels despite changes in gambling opportunities and availability.

Additionally, those whose financial status had been negatively affected by COVID-19 were more likely to feel their gambling had increased. Previous research has highlighted the negative relationship between financial insecurity and gambling (Auer, Malischnig, & Griffiths; Orford, 2004; Weinstein & Stone, 2018). Therefore, it is of concern that gambling may increase when an individual is faced with a downturn in economic circumstance. Alternative explanations are possible; those who found themselves working less may have more free time, and may have turned to gambling out of boredom. This is an area that warrants further exploration in subsequent studies.

### Limitations

Whilst providing some insight of the immediate influence of COVID-19 and lockdown on gambling behaviour in the UK, several limitations should be noted. The study only captured the first 4 weeks of lockdown; therefore, the longer-term impacts are as yet unknown. Inclusion/exclusion criteria were designed to be as inclusive as possible, but, this means the sample was not nationally representative. Furthermore, recruitment via crowdsourcing led to a proportion of participants who were non-gamblers, despite previous concerns that gamblers are overrepresented when recruiting via crowdsourcing (Mishra & Carleton, 2017; Schluter et al., 2018). Crowdsourcing was preferred over online advertising, due to the gambling-centric cohort available to the research team’s online study advertisement opportunities (i.e. social media) which may have biased results. Future studies could pre-screen for current gambling involvement to allow most efficient use of the sample. Additionally, this study relied on participants to accurately recollect events both pre- and during-lockdown and is therefore potentially subject to memory biases (Del Boca & Noll, 2000). Furthermore, unexpectedly, almost 75% of the sample were female; it is therefore unknown if the data in the current study are more representative of female gambling behaviours, or whether the findings apply to the gambling habits of the population more generally. Despite these limitations, the study nevertheless enabled the capture of a population of problematic, or potentially problematic, gamblers—who are now accessible for future study of further changes over time.

### Conclusions

The global COVID-19 pandemic and the subsequent Government response created a different environment for the UK public. This study explored the initial change in gambling behaviours in the UK, in the first weeks of lockdown. Results demonstrate that lockdown is not affecting the gambling behaviour of everyone in a uniform way, with those already most engaged with gambling maintaining that engagement despite the changing availability and accessibility of gambling. Whilst not claiming to provide definitive answers to how lockdown has affected gambling behaviour in the UK over the longer-term, this paper gives initial insight that provides a foundation for assessing and measuring the impact of COVID-19 on longer-term change in gambling behaviour.
